# Traffic exposure associated with allergic asthma and allergic rhinitis in adults. A cross-sectional study in southern Sweden

**DOI:** 10.1186/1476-072X-8-25

**Published:** 2009-05-06

**Authors:** Anna Lindgren, Emilie Stroh, Ulf Nihlén, Peter Montnémery, Anna Axmon, Kristina Jakobsson

**Affiliations:** 1 Department of Occupational and Environmental Medicine, Lund University, Sweden; 2 Astra Zeneca R&D, Lund, Sweden; 3 Department of Respiratory Medicine and Allergology, Lund University, Sweden; 4 Department of Community Medicine, Lund University, Sweden

## Abstract

**Background:**

There is conflicting evidence that traffic-related air pollution is a risk factor for allergic conditions. Few studies have investigated this in adults. In adults, a high proportion of asthma, rhinitis and eczema is triggered by non-allergic factors. We investigated traffic as a risk factor for allergic versus non-allergic asthma and rhinitis, and eczema, in adults.

A questionnaire from 2000 (n = 9319, 18–77 years) provided individual data about disease outcome and self-reported traffic exposure. Additional exposure assessments were obtained using Geographical Informations Systems (GIS). Residential addresses were linked to the national Swedish Road Database and to a pollutant database with modelled annual means of NO_x _(Nitrogen Oxids).

**Results:**

Living within 100 m from a road with a traffic intensity of >10 cars/min (24 hour mean) was associated with prevalence of current asthma reported to be triggered by allergic factors (OR = 1.83, 95% CI = 1.23–2.72) and with allergic rhinitis (OR = 1.30, 95%CI = (1.05–1.61). No relation was seen with asthma or rhinitis triggered by other factors. Living within 100 m of a road with >10 cars/min was also associated with hand-eczema during the last 12 months (OR = 1.63, 95% CI = 1.19–2.23), but not with allergic eczema or diagnosed hand-eczema. Consistent results were seen using self-reported traffic, but the associations with NO_x _were less consistent.

**Conclusion:**

Exposure to traffic was associated with a higher prevalence of allergic asthma and allergic rhinitis, but not with asthma or rhinitis triggered by non-allergic factors. This difference was suggested by the overall pattern, but only clear using GIS-measured traffic intensity as a proxy for traffic exposure. An association was also found with hand-eczema during the last 12 months. We suggest that asthma and rhinitis should not be treated as homogenous groups when estimating effects from traffic in adults.

## Background

There has been a significant increase in chronic respiratory diseases and allergy during the last decades. Air pollution from traffic has been one proposed risk factor. There is now evidence for long-term negative effects on lung function development [1], asthma [2], and COPD [3,4], but effects on allergic rhinitis and atopic dermatitis have remained unclear, even if a recent cohort study in children supports adverse effects [5].

An increased risk of asthma, allergic rhinitis, and eczema in individuals with a susceptibility for allergy (atopy) is well established [6], and it has been suggested that traffic pollution would increase or induce sensitivity for allergens in atopic individuals [7]. Support for this "sensitisation theory" stems mainly from laboratory studies [7], while epidemiologic studies estimating long-term effects on allergic conditions have shown conflicting results [8].

Traffic pollutions may potentiate allergic reactions in different ways [9]:

1) By attaching to the surface of e.g. pollen grains, air pollutants can change their morphology and enhance allergenic potential. 2) by inducing inflammation, which increases epithelial permeability, pollutants overcome the mucosal barrier and facilitate the allergen-induced inflammatory responses 3) diesel exhaust emissions increases immunoglobulin E synthesis, the dominating immune response in atopic subjects. Experimental studies have also shown that exposure to traffic-related air pollution can cause trans-epidermal water-loss [10] and decreased skin wheal response [11], in patients with atopic dermatitis.

Allergic symptoms often arise in childhood, and a majority of epidemiologic studies investigating effects from traffic on asthma, rhinitis and eczema have focused on children. In adults, a higher proportion of these diseases is triggered by non-allergic factors, than in children. Especially asthma is a heterogeneous condition in adults, and it has been suggested that asthma should not be used as a homogenous disease concept [12].

The present article is motivated by a previous study where we found asthma and COPD to be associated with traffic-related air pollution [13]. The present study investigates if both allergic and non-allergic subgroups of asthma are affected by traffic, and we also investigate the effect on allergic versus non-allergic rhinitis and eczema, in adults. GIS was used to complement self-reported traffic with external road data and a pollutant database for NO_x_, objective indicators for traffic-related air pollution at a local level.

## Materials and methods

### Study area

The study area was the south western part of the county of Scania, Sweden. The study area has a population of 840000 out of Sweden's total population of 8.9 millions, and a population density of 170 inhabitants/km^2 ^(data from 2000). The majority of the population is living in six of the municipalities, the largest of which is Malmö, the third largest city in Sweden, with a population of 260000. A detailed description of the study area has previously been given [14]. In the geographical stratification of the present study, "Malmö" refers strictly to the city boundaries of Malmö, not the larger municipality.

Although pollutant levels in the region are low in an European context, they are higher than in most other parts of Sweden [15], due to a relatively higher population density, long-range transport of pollutants from the continent, and more extensive road- harbour- and ferry traffic.

### Study population, Questionnaire and Geocoding

In 2000, a questionnaire was sent to a total of 11 933 randomly selected individuals aged 18–77 and 9 319 (78%) answered [13]. The study population originated from two different study populations, 5039 individuals (response rate 71%) from a new random selection, and 4280 individuals (response rate 87%) constituting a follow-up group from an earlier selection [16]. The questionnaire was focused on respiratory symptoms, but also contained information about eczema, smoking habits, occupation, and self-reported living close to traffic. The full questionnaire has been published previously [16]. Residential addresses were geocoded by linking each individual's unique 10-digit personal identity code to a registry containing geographical coordinates of all residential addresses. For non-responder analysis, see earlier publications [13,16].

### Outcome measures

**Figure 1:**
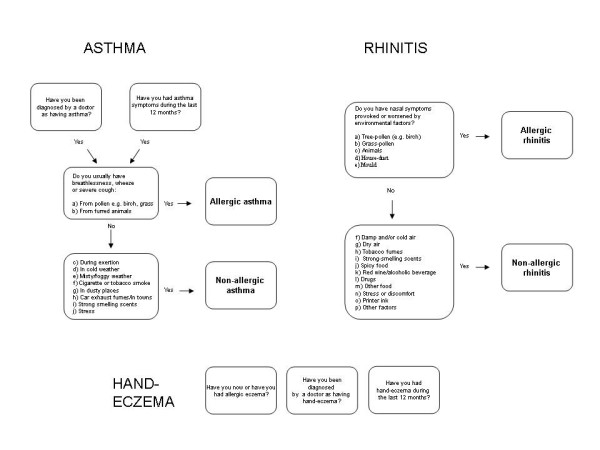
**Disease outcome definitions**.

Asthma, rhinitis and eczema were investigated using the questions specified in figure 1.

Current asthma was defined as self-reported physician diagnosed asthma in combination with asthma-symptoms last 12 months. This combination of questions has been validated in Sweden and showed a high specificity for asthma [17].

Subgroups of allergic versus non-allergic current asthma and rhinitis were defined by a question about what specific factors that usually triggered symptoms.

### Exposure assessment

Exposure to traffic was assessed at each participant's residential address in 2000, using three different proxies:

1. Self-reported exposure to traffic. This was obtained from the questionnaire. Exposure was defined as a positive answer to the question "*Do you live close to a road with heavy traffic?*".

2. Distance to roads with specified traffic intensity. GIS-based registers from *The Swedish National Road Database *[18] contained information about traffic intensity for all major roads in the county. To assess exposure to traffic, the individual was assigned the road with the heaviest traffic intensity within a radius of 100 m from residence. Traffic intensity was categorized as <2 cars/min, 2–5 cars/min, 6–10 cars/min and >10 cars/min, based upon annual 24 hour mean levels.

3. Modelled exposure to NO_x_. Annual mean concentrations of NO_x _were obtained from a GIS-based pollutant database for Scania based on the year 2001 [19]. Emission sources included in the model were: road traffic, shipping, aviation, railroad, industries and larger energy and heat producers, small scale heating, working machines, working vehicles and working tools. Meteorological data were also included. For dispersion calculations, a modified Gaussian dispersion model (AERMOD) was used, which is a flat two-dimensional model not adjusting for effects of street canyons or terrain, but taking the height of the emission sources into consideration. Concentrations of NO_x _were modelled as annual mean in a grid with a spatial resolution of 250 × 250 m. Bilinear interpolation was used to adjust individual exposure (based upon the individuals residence) with weighted values of neighbouring grid cells concentrations. Modelled concentrations with this spatial resolution have been validated and found to have a high correlation with measured values in the region [20-22].

### Potential confounders

For respiratory diseases, self-reported occupations were coded according to the European classification system ISCO-88 (COM), and the European job exposure matrix (JEM), ALOHA [23]. For eczema, a classification system based on risk occupations specifically for eczema was used [24]. Occupations were also coded according to the socio-economic indices (SEI-codes) officially used by Statistics Sweden [25].

### Statistics

Relative risk was estimated using Odds Ratios (OR:s) with 95% Confidence Intervals (CI). These were obtained by binary logistic regression, using SPSS, version 13.0. Sex, age and smoking (smokers and ex-smokers vs. non-smokers) were adjusted for in the model.

Occupational exposure and socio-economic status were tested as potential confounders. A predetermined change-in-estimate criteria of 10% would have motivated an inclusion in the model [26], but this was not fulfilled, neither was there any minor noticeable changes in estimate, why occupational exposure and socio-economic status were excluded from the model.

Odds ratios were not estimated in exposure groups with fewer than 50 individuals.

A categorical classification of NO_x _was used to be able to analyse non-linear associations between exposure to NO_x _and outcomes. To determine the category limits, the observations were merged and divided into NO_x_-quintiles. The five exposure groups used were 0–8 μg/m^3^, 8–11 μg/m^3^, 11–14 μg/m^3^, 14–19 μg/m^3^, and above 19 μg/m^3 ^.

NO_x _was also used as a continuous variable for trend analysis using logistic regression. A p-value < 0.05 was regarded as evidence of a trend.

Since areas with high exposure to traffic mainly were located in the city of Malmö, a geographical stratification (Malmö versus region outside Malmö) was done to exclude confounding from direct urban-rural comparison, when comparing high and low exposure.

We also investigated potential effect modification by stratified analysis on sex and smoking (current, former, never smoker).

In addition to current asthma, physician diagnosed asthma and asthma symptoms last 12 months were assessed separately in allergic vs non-allergic subgroups, to increase comparability with the previous study [13]. 72 of those with physician diagnosed asthma and 68 of those with asthma symptoms during the last 12 months had not reported any triggers and were therefore missing in the analysis.

## Results

Description of the relation between disease outcomes and covariates are given in table 1.

**Table 1:** Descriptives of study population, and disease prevalence in relation to sex, age, and smoking.

			Current asthma		Rhinitis		Eczema		
		Total n	Allergic	Non-allergic	Allergic	Non-allergic	Allergic eczema	Diagnosis of Hand-eczema	Hand-eczema last 12 months
Sex	Men	4341	106(2.4%)	57(1.3%)	800(18.4%)	266(6.1%)	326(7.5%)	171 (3.9%)	195 (4.5%)
	Women	4975	218(4.4%)	91(1.8%)	1064(21.4%)	339(8.0%)	813(16.3%)	430 (8.6%)	401 (8.1%)
									
Ever smoker	No	4306	143(3.3%)	53(1.2%)	941(21.9%)	254(5.9%)	504(11.7%)	245 (5.7%)	248 (5.8%)
	Yes	5010	181(3.6%)	95(1.9%)	923(18.4%)	351(7.0%)	635(12.7%)	356 (7.1%)	348 (6.9%)
									
Age	18–19	135	5(5.2%)	7(5.2%)	31(23%)	4(3.0%)	28(20.7%)	5 (3.7%)	3 (2.2%)
	20–29	1062	52(4.9%)	19(1.8%)	284(26.7%)	53(5.0%)	230(21.7%)	59 (5.6%)	80 (7.5%)
	30–39	2045	92(4.5%)	21(1.0%)	520(25.4%)	106(5.2%)	306(15.0%)	141 (6.9%)	166 (8.1%)
	40–49	1887	61(3.2%)	24(1.3%)	407(21.6%)	118(6.3%)	236(12.5%)	131 (6.9%)	132 (7.0%)
	50–59	2123	62(2.9%)	31(1.5%)	344(16.2%)	166(7.8%)	207(9.8%)	151 (7.1%)	134 (6.3%)
	60–69	1586	38(2.4%)	33(2.1%)	21813.7%)	122(7.7%)	112(7.1%)	94 (5.9%)	71 (4.5%)
	70–77	478	14(2.9%)	13(2.7%)	60(12.6%)	36(7.5%)	20(4.2%)	20 (4.2%)	10 (2.1%)

For description of reported triggers see additional file 1: Description of overlap between reported triggers of asthma and rhinitis.

In a stratified analysis, we found no evidence of effect modification by sex or smoking for any of the outcomes, although the power was also low to test for interaction.

### Asthma triggered by pollen or furred animals

Current asthma with symptoms reported to be triggered by pollen or furred animals, here defined as allergic asthma, was associated with self-reported traffic exposure and GIS-measured traffic intensity on heaviest road within 100 m, but not with modelled concentrations of NO_x _(table 2).

**Table 2:** Current asthma in relation to traffic.

		Current asthma					
		Allergic^a^			Non-allergic^b^		
		n^c^	n, %	Adj OR^d^	n^c^	n, %	Adj OR^d^
							
Heavy traffic	No	5441	187(3.4%)	1.00	5341	87(1.6%	1.00
	Yes	2881	137(4.8%)	1.32(1.05–1.66)	2805	61(2.2%)	1.28(0.92–1.79)
							
Heaviest road radie <100 m	no heavy road	3371	117(3.5%)	1.00	3316	62(1.9%)	1.00
	<2 cars/min	2014	79(3.9%)	1.13(0.84–1.51)	1966	31(1.6%)	0.82(0.53–1.28)
	2–5 cars/min	1608	54(3.4%)	0.96(0.69–1.33)	1584	30(1.9%)	0.98(0.63–1.53)
	6–10 cars/min	781	37(4.7%)	1.34(0.92–1.96)	759	15(2.0%)	0.95(0.54–1.69)
	>10 cars/min	511	35(6.8%)	1.83(1.23–2.72)	485	9(1.9%)	0.96(0.47–1.96)
							
NOx (ug/m3)	0–8	1665	68(4.1%)	1.00	1624	27(1.7%)	1.00
	8–11	1669	70(4.2%)	1.04(0.74–1.46)	1630	31(1.9%)	1.13(0.67–1.91)
	11–14	1661	52(3.1%)	0.74(0.51–1.07)	1641	32(2.0%)	1.15(0.69–1.94)
	14–19	1674	51(3.0%)	0.73(0.50–1.05)	1655	32(1.9%)	1.05(0.62–1.76)
	>19	1616	81(5.0%)	1.15(0.82–1.61)	1560	25(1.6%)	0.91(0.52–1.58)
							
			p-trend	0.669		p-trend	0.553

^a ^Asthma triggered by pollen or furred animals

^b^Asthma triggered by other factors

^c ^Individuals with non-allergic current asthma were excluded from the analysis of allergic current asthma in relation to traffic, and vice versa.

^d ^OR:s, 95% CI. Adjusted for age, sex and smoking

A geographical stratification showed increased prevalence in association with NO_x_, in Malmö, but not in the region outside (table 3). The association with self-reported traffic and GIS-measured traffic intensity seemed consistent across study area.

**Table 3:** Geographical stratification.

		Current asthma, allergic^a^					
		Malmö			Region outside Malmö		
		n^b^	n, %	Adj OR^c^	n^b^	n, %	Adj OR^c^

Heavy traffic	No	1586	55(3.5%)	1.00	3768	128(3.4%)	1.00
	Yes	1641	76(4.6%)	1.22(0.85–1.75)	1189	57(4.8%)	1.38(1.00–1.91)
							
Heaviest road radie <100 m (cars/min)	No road	517	16(3.1%)	1.00	2815	100(3.6%)	1.00
	<2	917	32(3.5%)	1.15(0.62–2.12)	1077	46(4.3%)	1.19(0.83–1.71)
	2–5	740	25(3.4%)	1.08(0.57–2.05)	847	27(3.2%)	0.89(0.58–1.37)
	6–10	581	26(4.5%)	1.49(0.79–2.82)	189	11(5.8%)	1.66(0.87–3.18)
	>10	472	32(6.8%)	1.96(1.05–3.66)	29	1	-
							
NOx (ug/m3)	0–8	12	0	-	1635	67(4.1%)	1.00
	8–11	43	4	-	1612	65(4.0%)	0.99(0.70–1.41)
	11–14	499	13(2.6%)	1.00	1138	38(3.3%)	0.79(0.52–1.19)
	14–19	1197	36(3.0%)	1.12(0.59–2.14)	457	14(3.1%)	0.74(0.41–1.33)
	>19	1476	78(5.3%)	1.78(0.97–3.27)	115	1(0.9%)	0.20(0.03–1.43)
							
			p-trend	0.019		p-trend	0.029

Current allergic asthma in the city of Malmö and the region outside.

^a ^Asthma triggered by pollen or furred animals

^b ^Individuals with non-allergic current asthma were excluded from the analysis of allergic current asthma in relation to traffic.

^c ^OR:s, 95% CI, Adjusted for age, sex and smoking

Separate assessment of asthma diagnosis and asthma symptoms during the last 12 months, triggered by allergic factors, showed the same patterns of associations with traffic as allergic current asthma (See additional file 2: Allergic vs. non-allergic physician-diagnosed asthma and asthma symptoms last 12 months).

### Asthma triggered by other factors

Current asthma triggered by non-allergic factor, was not associated with any of the exposure metrics (table 2).

A geographical stratification found no indications of effect modification by study area (table 4). Separate assessment of asthma diagnosis and asthma symptoms during the last 12 months, triggered by non- allergic factors, showed no association with traffic (See additional file 2: Allergic vs. non-allergic physician-diagnosed asthma and asthma symptoms last 12 months).

**Table 4:** Geographical stratification. Current non-allergic asthma in the city of Malmö and the region outside.

		Current asthma, non-allergic^a^					
		Malmö			Region outside Malmö		
		n^b^	n, %	Adj OR^c^	n^b^	n, %	Adj OR^c^

Heavy Traffic	No	1557	26(1.7%)	1.00	3700	60(1.6%)	1.00
	Yes	1599	34(2.1%)	1.31(0.78–2.21)	1159	27(2.3%)	1.37(0.86–2.17)
							
Heaviest road radie <100 m (cars/min)	No heavy road	512	11(2.1%)	1.00	2766	51(1.8%)	1.00
	<2 cars/min	902	17(1.9%)	0.88(0.41–1.89)	1045	14(1.3%)	0.73(0.40–1.33)
	2–5 cars/min	726	11(1.5%)	0.73(0.31–1.70)	839	19(2.3%)	1.17(0.68–2.00)
	6–10 cars/min	567	12(2.1%)	0.94(0.41–2.17)	181	3(1.7%)	0.82(0.25–2.66)
	>10 cars/min	449	9(2.0%)	1.00(0.41–2.46)	28	0	-
							
NOx (ug/m3)	0–8	12	0	-	1595	27(1.7%)	1.00
	8–11	39	0	-	1578	31(2.0%)	1.15(0.68–1.95)
	11–14	501	15(3.0%)	1.00	1117	17(1.5%)	0.86(0.46–1.59)
	14–19	1181	20(1.7%)	0.51(0.26–1.02)	455	12(2.6%)	1.50(0.75–3.00)
	>19	1423	25(1.8%)	0.58(0.30–1.12)	114	0(0%)	-
							
			p-trend	0.501		p-trend	0.677

^a^Asthma triggered by other factors

^b ^Individuals with allergic current asthma were excluded from the analysis of non-allergic current asthma in relation to traffic.

^c ^OR:s, 95% CI, Adjusted for age, sex and smoking

### Rhinitis triggered by pollen, furred animals, house dust or mould

Rhinitis triggered by pollen, animals, house dust or mould, was associated with all measures (table 5). A geographical stratification found no indications of effect modification by study area.

**Table 5:** Rhinitis in relation to traffic.

		Rhinitis					
		Allergic rhinitis^a^			Non-allergic rhinitis^b^		
		n^c^	n, %	Adj OR^d^	n^c^	n, %	Adj OR^d^
							
Heavy traffic	No	5641	1154(20.5%)	1.00	4887	400(8.2%)	1.00
	Yes	3070	710(23.1%)	1.13(1.01–1.26)	2565	205(8.0%)	0.99(0.83–1.18)
							
Heaviest road radie <100 m	no heavy road	3523	715(20.3%)	1.00	3040	232(7.6%)	1.00
	<2 cars/min	2087	421(20.2%)	0.99(0.87–1.14)	1814	148(8.2%)	1.08(0.87–1.34)
	2–5 cars/min	1684	373(22.1%)	1.11(0.96–1.28)	1447	136(9.4%)	1.27(1.01–1.58)
	6–10 cars/min	835	201(24.1%)	1.27(1.06–1.53)	685	51(7.4%)	0.96(0.70–1.32)
	>10 cars/min	544	143(26.3%)	1.30(1.05–1.61)	435	34(7.8%)	1.07(0.73–1.56)
							
NOx (ug/m3)	0–8	1759	329(18.7%)	1.00	1526	96(6.3%)	1.00
	8–11	1729	391(22.6%)	1.30(1.10–1.53)	1464	126(8.6%)	1.39(1.05–1.83)
	11–14	1731	368(21.3%)	1.16(0.98–1.38)	1487	124(8.3%)	1.37(1.04–1.80)
	14–19	1721	347(20.2%)	1.14(0.96–1.35)	1511	137(9.1%)	1.47(1.12–1.93)
	>19	1733	418(24.1%)	1.33(1.13–1.57)	1433	118(8.2%)	1.37(1.03–1.81)
							
			p-trend	0.006		p-trend	0.057

^a ^Rhinitis triggered by pollen, furred animals, house-dust or mould

^b^Rhinitis triggered by other factors

^c ^Individuals with non-allergic rhinitis were excluded from the analysis of allergic rhinitis in relation to traffic, and vice versa.

^d ^OR:s, 95% CI. Adjusted for age, sex and smoking

### Rhinitis triggered by other factors

Rhinitis triggered by non-allergic factors was not associated with self-reported traffic or GIS-measured traffic intensity, but showed a relation with modelled concentrations of NO_x _(table 5). A geographical stratification found no indication of effect modification by study area.

### Eczema

Self-reported allergic eczema was significantly associated with self-reported living close to a road with heavy traffic, and showed non-significant tendencies to a relation with the other measures. Self-reported physician diagnosed hand-eczema showed weak, but statistically non-significant, associations with traffic, while hand-eczema during the last 12 months showed a significant relation with self-reported living close to a road with heavy traffic and GIS-measured traffic intensity within 100 m, but not with modelled concentrations of NO_x _(table 6).

**Table 6:** Eczema in relation to traffic.

		Self-reported allergic eczema			Diagnosed hand-eczema		Hand-eczema last 12 months	
		n	n, %	Adj OR^a^	n, %	Adj OR^a^	n, %	Adj OR^a^
								
Heavy traffic	No	6041	681(11.3%)	1.00	373(6.2%)	1.00	345(5.7%)	1.00
	Yes	3275	458(14.0%)	1.16(1.02–1.32)	228(7.0%)	1.12(0.94–1.33)	251(7.7%)	1.32(1.12–1.57)
								
Heaviest road radie <100 m	no heavy road	3755	442(11.8%)	1.00	228(6.1%)	1.00	221(5.9%)	1.00
	<2 cars/min	2235	262(11.7%)	0.99(0.84–1.17)	148(6.6%)	1.10(0.89–1.37)	135(6.0%)	1.04(0.83–1.29)
	2–5 cars/min	1820	226(12.4%)	1.04(0.87–1.24)	116(6.4%)	1.08(0.86–1.37)	117(6.4%)	1.11(0.88–1.40)
	6–10 cars/min	886	119(13.4%)	1.15(0.92–1.43)	64(7.2%)	1.20(0.90–1.61)	65(7.3%)	1.29(0.97–1.72)
	>10 cars/min	578	84(14.5%)	1.08(0.83–1.40)	45(7.8%)	1.35(0.96–1.89)	56(9.7%)	1.63(1.19–2.23)
								
NOx (ug/m3)	0–8	1855	209(11.3%)	1.00	108(5.8%)	1.00	111(6.0%)	1.00
	8–11	1855	206(11.1%)	0.99(0.80–1.22)	124(6.7%)	1.15(0.88–1.50)	108(5.8%)	0.97(0.74–1.28)
	11–14	1855	251(13.5%)	1.19(0.98–1.46)	124(6.7%)	1.17(0.90–1.53)	131(7.1%)	1.20(0.92–1.56)
	14–19	1858	225(12.1%)	1.09(0.89–1.33)	123(6.6%)	1.15(0.88–1.50)	117(6.3%)	1.08(0.83–1.42)
	>19	1851	242(13.1%)	1.06(0.87–1.30)	122(6.6%)	1.16(0.89–1.52)	127(6.9%)	1.13(0.86–1.47)
								
			p-trend	0.44	p-trend	0.52	p-trend	0.357

^a ^OR:s, 95% CI, Adjusted for age, sex and smoking

A geographical stratification found no indications of effect modification by study area for allergic eczema, but some inconsistencies across study area for diagnosed hand-eczema and hand-eczema last 12 months. These inconsistencies were seen for all three measures but showed no consistent pattern (data not shown).

## Discussion

This study found traffic to be associated with higher prevalence of allergic asthma and allergic rhinitis, but not with non-allergic asthma and only with NO_x _for non-allergic rhinitis. The difference between allergic and non-allergic outcomes was suggested by overall pattern, but only clear using GIS-measured traffic intensity as a proxy for traffic exposure. An increased prevalence in relation to traffic was also seen on hand-eczema during the last 12 months.

### Study strengths and limitations

An important strength of the study was the use of three different proxies for exposure to traffic with high-quality of road- and emission data, and detailed questions of respiratory symptoms, which allowed for a distinction between allergic and non-allergic subjects. Symptoms triggered by pollen or furred animals can probably be seen as highly specific for allergy. However, "symptoms triggered by other factors" is a heterogenous grouping, and these results should be interpreted with caution. It should be noted that only trigger-dependent symptoms were analysed in this study, not non-allergic chronic respiratory symptoms which are not dependent on triggers.

Self-report of allergic triggers has shown moderate association with skin prick-test [27], but this association does not necessarily reflect the validity of self-report, but also reflects that not all which show positive prick-test have actual symptoms of their allergy. While about 40% of the western population have elevated levels of IgE to common environmental allergens, only about 7% express their atopy as asthma [28]. Since air pollution might exert effects either in sensitization or in later manifestation of disease, biological markers should be related to reports and tests of actual symptoms. Our study strongly indicates that allergic asthma and allergic rhinitis are affected by traffic in adults, but the lack of biological markers and objective symptom testing is a limitation.

A limitation was also the cross-sectional study design, which makes it difficult to assess if pollution is associated with the onset of allergy or only trigger an existing allergic disease.

We had no possibility to properly assess retrospective exposure. We therefore focused on current asthma since symptoms last 12 months are in agreement with estimated exposure, and ever doctor's diagnosis exclude asthmatic symptoms not specific of asthma.

Even if the additional separate association with ever diagnosis of asthma indicates long-term effects, there is a possibility of recall-bias, where those with current symptoms are more likely to remember being diagnosed, which would bias these effects away from null. On the other hand, since asthma and rhinitis could be triggered by traffic pollution, those with respiratory symptoms are also likely to be affected by migrational bias, which would rather bias both the effects of diagnosis and current symptoms towards null.

The traffic exposure measures have been more thoroughly discussed in a related article [13]. Self-reported traffic mainly showed consistent, although less pronounced results compared with using GIS-measured traffic intensity. The GIS-based road proxy has the advantage to not be limited by spatial aggregation, but is a simple proxy for exposure, only considering the heaviest road within a certain radius. Modelled levels of NO_x _on the other hand, takes total traffic density into account, but had the disadvantage to be the measure with the lowest spatial resolution, and may therefore be most sensitive for ecological bias. The finding that associations with NO_x _for allergic asthma were only seen in Malmö, may indicate unmeasured confounding and/or that NO_x _is not a good proxy of traffic-related air pollution outside urban areas, something we have discussed in a previous article where we analysed asthma as a homogenous group [13].

### Discussion of main results and comparison with other studies

There was a clear relation between exposure to traffic and asthma triggered by pollen or furred animals, but not with asthma triggered by other factors. This result seems to be supported by a Swedish study which found that an increased incidence of adult asthma associated with increase in NO_2_only occurred among atopics [29]. The Swedish cities in the RHINE-study however, found no interaction between asthma and NO_2 _using hay-fever as a proxy for atopy [30]. The ECRHS-study also found no interaction with atopy for the relation between traffic and adult asthma incidence [31], and no relation between traffic and sensitization [32]. The Swiss SAPALDIA study found traffic to be related to allergic sensitization to pollen in skin prick-test, but not with asthma symptoms, at baseline [33]. In the recently published follow-up, those with atopy at baseline seemed to have a higher incidence of asthma in relation to traffic, although there was not enough power for statistical confirmation [34]. A German study found neither increase of asthma or allergic sensitization living at self-reported busy roads [35]. Comparison with our study is complicated by the fact that atopy could both act as effect-modifier and mediator to disease. None of the abovementioned studies have directly related traffic to allergic asthma.

Consistent with the results for asthma, rhinitis due to pollen or furred animals were affected by traffic, but not rhinitis triggered by other factors, which showed an association with NO_x_, but no convincing overall trend toward a relation with traffic. There is previous weak epidemiologic support for an effect from traffic on allergic rhinitis in adults. The Swiss SAPALDIA study in 2000 found living close to busy roads not to be associated with allergic rhinitis [33]. In Germany, living close to extremely or considerably busy roads has been associated with an marginally increased risk of allergic rhinitis (OR = 1.16 (0.94–1.42) [35]. An Italian study in adults found outdoor NO_2 _exposure to be associated with significantly increased prevalence of allergic rhinitis in the Mediterranean region (OR = 1.38; 95% CI 1.12 to 1.69), but not in the subcontinental region, and concluded that climate interacts with effects of NO_2 _outdoor exposure [36]. Our results strengthens previous very weak evidence for associations between traffic and self-reported allergic rhinitis in adults, but it should be noted that the specific question we used for definition of allergic rhinitis differs from what has been used in other studies.

There was a higher prevalence of allergic eczema and hand-eczema in relation to heavy traffic, but this was only significant for self-reported hand-eczema during the last 12 months. It had been desirable to make a distinction between atopic dermatitis and contact eczema, but this distinction has low validity in questionnaires without clinical examination or validated differential questions, such as debut of hand-eczema in childhood or presence of nickel allergy [37]. Occupational exposure is a major risk factor for hand-eczema, but was not found to be a confounder with the present assessment of risk occupations. Since Sweden has a largely segregated labour market in respect of gender [38], adjustment for sex and age may partly adjust for risk occupation. Few epidemiological studies have investigated the effect from traffic on atopic dermatitis. A previous cross-sectional study in southern Sweden in 1992, related to this study, found self-reported traffic to be associated with allergic eczema (OR = 1.45, 95% CI 1.28–1.66), but this seems to be the only evidence of effects of traffic on eczema in adults. In children, a few studies have indicated long-term effects on atopic dermatitis [5,39,40]. To our knowledge, no epidemiologic study has previously studied effects from traffic on hand-eczema.

In conclusion, the present study of a randomly selected adult population found that allergic asthma and allergic rhinitis are associated with traffic-related air pollution, but not non-allergic asthma or rhinitis. This result suggests that asthma and rhinitis should be divided into allergic and non-allergic conditions when investigating effects from traffic pollution in adults. However, the cross-sectional design is a severe limitation of this study, and longitudinal studies in adults are needed to investigate if the effects for allergic versus non-allergic chronic respiratory disease reflects adult onset disease. Potential biological mechanisms can also not be explained in our epidemiological study, which lacked biological markers, but the indications of effects on eczema are interesting and either indicate that adverse effects from traffic on allergic disease are not limited to the respiratory tract, or that exposure to traffic have negative effects on the skin which are not related to allergic disease.

## Conclusion

This study found that exposure to traffic is associated with a higher prevalence of allergic asthma and allergic rhinitis, but not with asthma or rhinitis triggered by non-allergic factors. This difference was suggested by the overall pattern, but only clear using GIS-measured traffic intensity as a proxy for traffic exposure. An association was also found with hand-eczema. We suggest that asthma and rhinitis should not be treated as homogenous groups when estimating effects from traffic in adults.

## Competing interests

The authors declare that they have no competing interests.

## Authors' contributions

AL: Decided the content of the article, conducted the statistical analyses and wrote the main part of the manuscript. ES: Performed GIS analyses and wrote part of the manuscript. UN: Designed and conducted the survey and made revisions on draft. PM: Designed and conducted the survey and made revisions on draft. AA: Made revisions on draft. KJ: Made major revisions on draft. All authors read and approved the final manuscript.

## Supplementary Material

Additional file 1 **Description of overlap between reported triggers of asthma and rhinitis.** Click here for file

Additional file 2 **Allergic vs. non-allergic physician-diagnosed asthma and asthma symptoms last 12 months.** Click here for file
