# Supplementary material for: Traffic exposure associated with allergic asthma and allergic rhinitis in adults. A cross-sectional study in southern Sweden
Source: Int J Health Geogr. 2009 May 6;8:25. doi: 10.1186/1476-072X-8-25 (PMC2687434; doi:10.1186/1476-072X-8-25)
Supplement: Additional file 1 — Description of overlap between reported triggers of asthma and rhinitis. [file 1476-072X-8-25-S1.pdf]

Asthma triggers. Description of overlap between reported triggers of asthma, among those with current asthma.

Counts and (%) of row total. The first row shows that 52% of those with current asthma triggered by pollen had also reported asthma triggered by furred animals 73% of those with asthma triggered by pollen had reported asthma triggered by exertion, etc.

	Total n	Pollen	Furred animals	During Exertion	Cold weather	Foggy weather	Tobacco smoke	Dusty places	Car exhaust	Strong scents	Stress
Pollen	269	-	139 (52)	195 (73)	143 (53)	159 (59)	164 (61)	130 (48)	104 (39)	173 (64)	106 (39)
Furred animals	194	139 (72)	-	141 (73)	110 (57)	115 (59)	121 (62)	89 (46)	70 (36)	119 (61)	74 (38)
During exertion	355	195 (55)	141 (40)	-	206 (58)	221 (62)	218 (61)	171 (48)	139 (39)	204 (58)	138 (39)
Cold weather	250	143 (57)	110 (44)	206 (82)	-	171 (68)	156 (62)	122 (49)	101 (40)	157 (63)	101 (40)
Foggy weather	262	159 (61)	115 (44)	221 (84)	171 (65)	-	177 (68)	171 (65)	120 (46)	169 (65)	120 (46)
Tobacco smoke	267	164 (61)	121 (45)	218 (82)	156 (58)	177 (66)	-	153 (57)	142 (53)	179 (67)	107 (40)
Dusty places	201	130 (65)	89 (44)	171 (85)	122 (61)	171 (85)	153 (76)	-	111 (55)	142 (71)	101 (50)
Car exhaust	170	104 (61)	70 (41)	139 (82)	101 (59)	120 (71)	142 (84)	111 (65)	-	130 (77)	87 (51)
Strong scents	267	173 (65)	119 (45)	204 (76)	157 (59)	169 (63)	179 (67)	142 (53)	130 (49)	-	110 (41)
Stress	166	106 (64)	74 (45)	138 (83)	101 (61)	120 (72)	107 (65)	101 (61)	87 (52)	110 (66)	-

Rhinitis triggers. Description of overlap between reported triggers of rhinitis.

Counts and (%) of row total. The first row shows that 80% of those with rhinitis triggered by tree pollen had also reported rhinitis triggered by grass pollen. 37% of those with rhinitis triggered by tree pollen had reported rhinitis triggered by furred animals.

	n	Tree pollen	Grass pollen	Furred animals	House-dust	Mould	Damp/ cold air	Dry air	Tobacco	Strong scents	Spicy food	Red wine /alcohol	Other food	Drugs	Stress	Printers ink	Other factors
Tree pollen	981	-	781 (80)	358 (37)	393 (40)	255 (26)	211 (22)	194 (20)	330 (34)	400 (41)	130 (13)	118 (12)	103 (11)	29 (3)	112 (11)	187 (19)	85 (9)
Grass pollen	1171	781 (67)	-	426 (36)	468 (40)	300 (26)	263 (23)	246 (21)	388 (33)	454 (39)	150 (13)	135 (12)	97 (8)	30 (3)	143 (12)	219 (19)	89 (8)
Furred animals	613	358 (58)	426(70)	-	326 (53)	193 (32)	163 (27)	129 (21)	258 (42)	278 (45)	78 (13)	82 (13)	79 (13)	22 (4)	74 (12)	128 (21)	49 (8)
House-dust	870	393 (45)	468 (54)	326 (38)	-	314 (36)	267 (31)	253 (29)	410 (47)	407 (47)	157 (18)	120 (14)	69 (8)	25 (3)	143 (16)	206 (24)	86 (10)
Mould	527	255 (48)	300 (57)	193 (37)	314 (60)	-	223 (42)	180 (34)	279 (53)	286 (54)	100 (19)	87 (17)	58 (11)	27 (5)	108 (21)	156 (30)	63 (12)
Damp/cold air	613	211 (34)	263 (43)	163 (27)	267 (44)	223 (36)	-	199 (33)	278 (45)	263 (43)	137 (22)	93 (15)	49 (8)	24 (4)	146 (24)	135 (22)	66 (11)
Dry air	610	194 (32)	246 (40)	129 (21)	253 (42)	180 (30)	199 (33)	-	301 (49)	285 (47)	92 (15)	106 (17)	37 (6)	20 (3)	115 (19)	134 (22)	60 (10)
Tobacco	823	330 (40)	388 (47)	258 (31)	410 (50)	279 (34)	278 (34)	301 (37)	-	462 (56)	143 (17)	135 (16)	71 (9)	26 (3)	152 (19)	204 (25)	76 (9)
Strong scents	874	400 (46)	454 (52)	278 (32)	407 (47)	286 (33)	263 (30)	285 (33)	462 (53)	-	176 (20)	156 (18)	77 (9)	30 (3)	142 (16)	260 (30)	96 (11)
Spicy food	314	130 (41)	150 (48)	78 (25)	157 (50)	100 (32)	137 (44)	92 (29)	143 (46)	176 (56)	-	77 (25)	38 (12)	15 (5)	77 (25)	91 (29)	42 (13)
Red wine/alcohol	316	118 (37)	135 (43)	82 (26)	120 (38)	87 (28)	93 (30)	106 (34)	135 (43)	156 (49)	77 (24)	-	36 (11)	12 (4)	59 (19)	95 (30)	36 (11)
Other food	149	103 (69)	97(65)	79 (53)	69 (46)	58 (39)	49 (33)	37 (25)	71 (48)	77 (52)	38 (26)	36 (24)	-	17 (11)	27 (18)	40 (27)	22 (15)
Drugs	56	29 (52)	30 (54)	22 (39)	25 (45)	27 (48)	24 (43)	20 (36)	26 (46)	30 (54)	15 (27)	12 (21)	17 (30)	-	14 (25)	22 (39)	11 (20)
Stress	332	112 (34)	143 (43)	74 (22)	143 (43)	108 (33)	146 (44)	115 (35)	152 (46)	142 (43)	77 (23)	59 (18)	27 (8)	14 (4)	-	77 (23)	35 (11)
Ink	444	187 (42)	219 (49)	128 (29)	206 (46)	156 (35)	135 (30)	134 (30)	204 (46)	260 (59)	91 (21)	95 (21)	40 (9)	22 (5)	77 (17)	-	61 (14)
Other factor	303	85 (28)	89 (29)	49 (16)	86 (28)	63 (21)	66 (22)	60 (20)	76 (25)	96 (32)	42 (14)	36 (12)	22 (7)	11 (4)	35 (12)	61(20)	-
