# Supplementary material for: Traffic exposure associated with allergic asthma and allergic rhinitis in adults. A cross-sectional study in southern Sweden
Source: Int J Health Geogr. 2009 May 6;8:25. doi: 10.1186/1476-072X-8-25 (PMC2687434; doi:10.1186/1476-072X-8-25)
Supplement: Additional file 2 — Allergic vs. non-allergic physician-diagnosed asthma and asthma symptoms last 12 months. [file 1476-072X-8-25-S2.pdf]

Table 1. Allergic vs. nonallergic physician-diagnosed asthma and asthma symptoms last 12 months, in relation to traffic.

			Asthma diagnosis				Asthmasymptoms last 12 months			
			Allergic [a]		Non-allergic [b]		Allergic [a]		Non-allergic [b]	
		n	n, %	Adj OR [c]	n, %	Adj OR [c]	n, %	Adj OR [c]	n, %	Adj OR [c]
Heavy traffic	No	6041	224(3.7%)	1.00	132(2.2%)	1.00	343(5.7%)	1.00	284(4.7%)	1.00
	Yes	3275	169(5.2%)	1.33(1.08-1.64)	89(2.7%)	1.20(0.91-1.58)	240(7.3%)	1.25(1.05-1.48)	180(5.5%)	1.15(0.95-1.40)
Heaviest road within <100 m	No heavy road	3755	143(3.8%)	1.00	92(2.5%)	1.00	207(5.5%)	1.00	187(5.0%)	1.00
	<2 cars/min	2235	93(4.2%)	1.09(0.84-1.43)	43(1.9%)	0.77(0.53-1.11)	133(6.0%)	1.08(0.86-1.35)	113(5.1%)	1.00(0.79-1.27)
	2-5cars/min	1820	67(3.7%)	0.95(0.71-1.28)	55(3.0%)	1.20(0.85-1.69)	115(6.3%)	1.14(0.90-1.45)	86(4.7%)	0.94(0.72-1.22)
	6-10cars/min	886	44(5.0%)	1.30(0.92-1.84)	19(2.1%)	0.79(0.48-1.30)	70(7.9%)	1.44(1.09-1.91)	48(5.4%)	1.01(0.72-1.40)
	>10cars/min	578	43(7.4%)	1.83(1.28-2.62)	11(1.9%)	0.74(0.39-1.40)	54(9.3%)	1.62(1.18-2.22)	29(5.0%)	1.01(0.67-1.52)
NO_x ($\mu g/m^3$)	0-8	1855	79(4.3%)	1.00	42(2.3%)	1.00	115(6.2%)	1.00	91(4.9%)	1.00
	8-11	1855	84(4.5%)	1.07(0.78-1.46)	48(2.6%)	1.12(0.73-1.70)	113(6.1%)	0.99(0.75-1.29)	89(4.8%)	0.96(0.71-1.30)
	11-14	1855	62(3.3%)	0.75(0.54-1.06)	46(2.5%)	1.05(0.68-1.60)	104(5.6%)	0.88(0.67-1.16)	87(4.7%)	0.95(0.70-1.28)
	14-19	1858	64(3.4%)	0.80(0.57-1.11)	45(2.4%)	0.96(0.63-1.48)	105(5.7%)	0.90(0.69-1.19)	86(4.6%)	0.88(0.651-1.19)
	>19	1851	101(5.5%)	1.21(0.89-1.64)	39(2.1%)	0.87(0.55-1.35)	142(7.7%)	1.19(0.92-1.54)	110(5.9%)	1.20(0.90-1.61)

[a] Asthma triggered by pollen or furred animals [b]Asthma symptoms triggered by other factors [c] OR:s, 95% CI. Adjusted for age, sex and smoking

Table 2. Geographical stratification. Allergic physician-diagnosed asthma and asthma symptoms last 12 months, in relation to traffic.

		Asthma diagnosis, allergic [a]				Asthma symptoms last 12 months, allergic [a]			
		Malmö		Region outside Malmö		Malmö		Region outside Malmö	
		n, %	Adj OR [b]	n, %	Adj OR [b]	n, %	Adj OR [b]	n, %	Adj OR [b]
Heavy traffic	No	66(3.7%)	1.00	151(3.6%)	1.00	102(5.8%)	1.00	233(5.6%)	1.00
	Yes	98(5.2%)	1.29(0.93-1.78)	66(4.9%)	1.33(0.99-1.80)	133(7.1%)	1.16(0.89-1.52)	102(7.6%)	1.35(1.06-1.73)
Heaviest road within <100 m	No heavy road	22(3.8%)	1.00	117(3.7%)	1.00	33(5.6%)	1.00	171(5.5%)	1.00
	<2 cars/min	40(3.9%)	1.06(0.63-1.81)	52(4.4%)	1.15(0.82-1.61)	53(5.2%)	0.94(0.60-1.47)	78(6.5%)	1.18(0.90-1.56)
	2-5cars/min	32(3.8%)	1.00(0.58-1.75)	33(3.4%)	0.91(0.61-1.35)	50(6.0%)	1.07(0.68-1.69)	63(6.6%)	1.20(0.89-1.62)
	6-10cars/min	30(4.5%)	1.25(0.71-2.20)	14(6.6%)	1.78(1.00-3.18)	49(7.4%)	1.35(0.86-2.14)	21(9.9%)	1.86(1.15-3.00)
	>10cars/min	40(7.4%)	1.83(1.07-3.15)	1	-	50(9.3%)	1.59(1.00-2.53)	2	-
NO_x ($\mu g/m^3$)	0-8	0	-	78(4.3%)	1.00	0	-	114(6.3%)	1.00
	8-11	5	-	76(4.2%)	0.99(0.72-1.37)	5	-	106(5.9%)	0.95(0.73-1.26)
	11-14	19(3.4%)	1.00	42(3.3%)	0.75(0.51-1.10)	24(4.3%)	1.00	79(6.2%)	0.98(0.72-1.32)
	14-19	43(3.2%)	0.95(0.55-1.65)	19(3.7%)	0.87(0.52-1.10)	71(5.4%)	1.24(0.77-1.99)	32(6.3%)	1.01(0.67-1.52)
	>19	97(5.7%)	1.53(0.92-2.54)	2(1.6%)	0.34(0.08-1.42)	135(8.0%)	1.77(1.13-2.79)	4(3.1%)	0.46(0.17-1.28)

[a]Asthma triggered by pollen or furred animals [b] OR:s, 95% CI, Adjusted for age, sex and smoking

Table 3. Geographical stratification. Non-allergic physician-diagnosed asthma and asthma symptoms last 12 months, in relation to traffic.

		Asthma diagnosis, non-allergic [a]				Asthma symptoms last 12 months, non-allergic [a]			
		Malmö		Region outside Malmö		Malmö		Region outside Malmö	
		n, %	Adj OR [b]	n, %	Adj OR [b]	n, %	Adj OR [b]	n, %	Adj OR [b]
Heavy traffic	No	35(2.0%)	1.00	96(2.3%)	1.00	92(5.2%)	1.00	191(4.6%)	1.00
	Yes	48(2.6%)	1.33(0.85-2.08)	41(3.1%)	1.26(0.87-1.83)	114(6.1%)	1.19(0.89-1.59)	65(4.8%)	1.04(0.78-1.39)
Heaviest road within <100 m	No heavy road	15(2.6%)	1.00	77(2.5%)	1.00	35(6.0%)	1.00	152(4.9%)	1.00
	<2 cars/min	23(2.3%)	0.87(0.45-1.69)	20(1.7%)	0.69(0.42-1.13)	58(5.7%)	0.96(0.62-1.48)	55(4.6%)	0.95(0.69-1.31)
	2-5cars/min	19(2.3%)	0.90(0.45-1.78)	36(3.7%)	1.47(0.98-2.20)	44(5.3%)	0.91(0.57-1.43)	41(4.3%)	0.86(0.60-1.22)
	6-10cars/min	15(2.3%)	0.84(0.41-1.74)	4(1.9%)	0.69(0.25-1.92)	40(6.0%)	0.98(0.61-1.56)	8(3.8%)	0.72(0.35-1.50)
	>10cars/min	11(2.0%)	0.81(0.36-1.78)	0	-	29(5.4%)	0.95(0.57-1.58)	0	-
NO_x ($\mu g/m^3$)	0-8	0	-	42(2.3%)	1.00	0	-	91(5.0%)	1.00
	8-11	0	-	48(2.7%)	1.14(0.75-1.73)	1	-	88(4.9%)	0.96(0.71-1.30)
	11-14	17(3.0%)	1.00	29(2.3%)	0.92(0.57-1.49)	37(6.6%)	1.00	50(3.9%)	0.77(0.54-1.10)
	14-19	29(2.2%)	0.67/0.36-1.23)	16(3.1%)	1.29(0.72-2.33)	61(4.6%)	0.64(0.42-0.98)	24(4.7%)	0.94(0.59-1.49)
	>19	37(2.2%)	0.70(0.39-1.26)	2(1.6%)	0.65(0.16-2.74)	107(6.3%)	0.97(0.65-1.43)	3(2.4%)	0.48(0.15-1.55)

[a]Asthma triggered by “other factors” [b] OR:s, 95% CI, Adjusted for age, sex and smoking
